# Educational Level, Anticoagulation Quality, and Clinical Outcomes in Elderly Patients with Acute Venous Thromboembolism: A Prospective Cohort Study

**DOI:** 10.1371/journal.pone.0162108

**Published:** 2016-09-08

**Authors:** Eveline Hofmann, Nicolas Faller, Andreas Limacher, Marie Méan, Tobias Tritschler, Nicolas Rodondi, Drahomir Aujesky

**Affiliations:** 1 Department of General Internal Medicine, Bern University Hospital, University of Bern, Bern, Switzerland; 2 Clinical Trials Unit Bern, Department of Clinical Research, and Institute of Social and Preventive Medicine, University of Bern, Bern, Switzerland; 3 Department of General Internal Medicine, Lausanne University Hospital, Lausanne, Switzerland; Maastricht University Medical Center, NETHERLANDS

## Abstract

Whether the level of education is associated with anticoagulation quality and clinical outcomes in patients with acute venous thromboembolism (VTE) is uncertain. We thus aimed to investigate the association between educational level and anticoagulation quality and clinical outcomes in elderly patients with acute VTE. We studied 817 patients aged ≥65 years with acute VTE from a Swiss prospective multicenter cohort study (09/2009-12/2013). We defined three educational levels: 1) less than high school, 2) high school, and 3) post-secondary degree. The primary outcome was the anticoagulation quality, expressed as the percentage of time spent in the therapeutic INR range (TTR). Secondary outcomes were the time to a first recurrent VTE and major bleeding. We adjusted for potential confounders and periods of anticoagulation. Overall, 56% of patients had less than high school, 25% a high school degree, and 18% a post-secondary degree. The mean percentage of TTR was similar across educational levels (less than high school, 61%; high school, 64%; and post-secondary, 63%; *P* = 0.36). Within three years of follow-up, patients with less than high school, high school, and a post-secondary degree had a cumulative incidence of recurrent VTE of 14.2%, 12.9%, and 16.4%, and a cumulative incidence of major bleeding of 13.3%, 15.1%, and 15.4%, respectively. After adjustment, educational level was neither associated with anticoagulation quality nor with recurrent VTE or major bleeding. In elderly patients with VTE, we did not find an association between educational level and anticoagulation quality or clinical outcomes.

## Introduction

Given the narrow therapeutic range of vitamin K antagonists, a strict adherence to anticoagulant therapy is important in the management of venous thromboembolism (VTE). Supra-therapeutic anticoagulation, defined as an international normalized ratio (INR) >3.0, increases the risk of bleeding, whereas sub-therapeutic anticoagulation (INR <2.0) may increase the risk of recurrent VTE [1]. Socioeconomic factors, such as poverty or homelessness, were found to be associated with lower adherence to anticoagulation therapy [2].

The educational level, defined as the highest level of schooling reached, is an important socioeconomic factor and has substantial health consequences [3]. A low educational level continues to increase the risk of adverse health effects even among the elderly [4]. Although patients with a lower educational level are more likely to have limited language proficiency, health literacy, and lower drug adherence and warfarin knowledge scores [5–9], whether educational level is associated with anticoagulation quality in patients with VTE is uncertain. Prior studies examining this question were limited by a cross-sectional design [10, 11] or a small sample size [11–14], did not focus on patients with VTE [10–14], or assessed anticoagulation quality indirectly using self-reported drug compliance or electronic medication monitoring systems rather than the time spent in therapeutic INR range (TTR) [11, 14].

According to population-based registries, patients with a lower educational level appear to have an increased overall risk of VTE [15, 16]. However, whether a lower education is associated with recurrent VTE or anticoagulation-related bleeding in patients with acute VTE is unknown. To fill these gaps of knowledge, we evaluated the association between educational level and the quality of anticoagulation in a prospective multicenter cohort of elderly patients with acute VTE. We also examined whether the educational level was associated with recurrent VTE or major bleeding.

## Methods

### Cohort sample

The study was conducted between September 2009 and December 2013 as part of a prospective, multicenter cohort study (SWITCO65+) to asses long-term medical outcomes and quality of life in consecutive in- and outpatients aged 65 years or older with acute symptomatic, objectively confirmed VTE from all five Swiss university and four high-volume non-university hospitals [17]. The patient enrolment phase ended in March 2012 and patients were followed-up until December 2013. VTE comprised proximal and distal deep vein thrombosis (DVT) and/or pulmonary embolism (PE). Exclusion criteria were catheter-related thrombosis, thrombosis at a different site than lower limb, insufficient German or French-speaking ability, impossibility to follow up (i.e., terminal illness), an inability to provide informed consent (i.e., severe dementia), or previous enrollment in the cohort. The detailed study methods, including eligibility criteria and exact definitions of DVT and PE, were published previously [17]. The Institutional Review Board at each participating center approved the study and patients gave written consent to participation. The approving ethic committees were the “Commission cantonale d’éthique de la recherche sur l’être humain Vaud” (site of Lausanne), “Commission cantonale d'éthique de la recherche Genève” (site of Geneva), “Kantonale Ethikkommission Bern” (site of Bern), “Kantonale Ethikkommission Zürich” (site of Zurich), “Ethikkommission Nordwest- und Zentralschweiz” (sites of Basel, Lucerne and Baden), “Ethikkommission des Kantons Thurgau” (site of Frauenfeld) and “Ethikkommission des Kan- tons St. Gallen” (site of St. Gallen). For the present study, we considered all patients of the original cohort who were treated with vitamin K antagonists within 30 days of VTE diagnosis.

### Baseline data collection

For all enrolled patients, trained study nurses prospectively collected information about baseline demographics such as age, gender, living status (living at home with another person or alone, or living in a nursing home), and self-reported educational level. Additional data collection included smoking status, body mass index, average weekly alcohol consumption, recent major surgery, comorbid conditions (active cancer, arterial hypertension, chronic heart failure, diabetes mellitus, cerebrovascular disease, chronic liver disease, chronic renal failure, inflammatory bowel disease, history of VTE or major bleeding), localization of index VTE (DVT only, PE only, or both), type of VTE (provoked, unprovoked, or cancer-related), routine laboratory findings (hemoglobin, platelet count), risk of falls, concomitant antiplatelet therapy or non-steroidal anti-inflammatory drugs, polypharmacy. The risk of falls was assessed using two validated screening questions: 1) did you fall during the last year? and 2) did you notice any problem with gait, balance, or mobility [18]? Patients who answered yes to at least one screening question were considered to be at high risk of falls. Polypharmacy was defined as the prescription of more than four drugs, including St. John’s wort, at the time of the index VTE event [19]. The intake of vitamins or alternative medicine treatments was not considered.

### Level of education

Study nurses assessed the patient’s self-reported level of education at baseline. We defined three educational levels: 1) less than high school education (≤9 years of schooling completed), 2) high school degree (high school completed), or 3) post-secondary degree (diploma from a university or an equivalent institution), as done previously [15, 16].

### Anticoagulation management

Patients were treated with Acenocoumarol and Phenprocoumone, the two vitamin K antagonists available in Switzerland. Patients received discharge instructions and educational measures on anticoagulation by their managing physicians. After discharge, anticoagulation was managed by primary care physicians who determined the frequency of INR measurements on an individual basis.

### Study outcomes

The primary outcome of this study was the quality of anticoagulation, expressed as the percentage of time spent in the therapeutic range (TTR) of the INR (2.0–3.0) according to the Rosendaal method [20]. Secondary outcomes were clinical events, i.e. the time to a first recurrent VTE and major bleeding. Recurrent VTE was defined as a new or recurrent, fatal or non-fatal, symptomatic, and objectively confirmed PE or DVT, as previously described [17]. We defined major bleeding as a fatal bleeding, a symptomatic bleeding in a critical organ (intracranial, intraspinal, intraocular, retroperitoneal, intraarticular, pericardial, or intramuscular with compartment syndrome), a bleeding with a reduction of hemoglobin ≥20 g/l, or a bleeding leading to the transfusion of ≥2 units of packed red blood cells [21].

Follow-up included one telephone interview and two face-to-face evaluations during the first year of study participation and then semi-annual contacts, alternating between face to-face-evaluations and telephone calls as well as periodic hospital chart reviews. As part of the follow-up interview/visits, study nurses obtained information about the date and type of VTE recurrence, bleeding events, and death. We also collected INR values throughout follow-up. A committee of three blinded clinical experts adjudicated all outcomes. The committee classified the cause of all deaths as definitely due to PE (i.e., confirmed by autopsy or death followed a clinically severe PE), possibly due to PE (i.e., death in a patient who died suddenly or unexpectedly), due to bleeding, or due to another cause. Death was judged to be bleeding-related if it followed an intracranial hemorrhage or a bleeding episode leading to hemodynamic deterioration [22]. Final classifications were made on the basis of the full consensus of this committee.

### Statistical analysis

We compared patient baseline characteristics by educational level using the chi-squared and Kruskal-Wallis rank tests as appropriate. We compared the percentage of time spent within one of three specified INR ranges (<2.0, 2.0–3.0, >3.0) across educational levels using analysis of variance and adjusted regression models, excluding the first seven treatment days [20]. We compared the cumulative incidence of recurrent VTE and major bleeding by educational level using Kaplan-Meier analysis and the log rank test.

We examined the association between educational level and the TTR using linear regression models, adjusting for known risk factors of poor anticoagulation quality, including age, female gender, living status, body mass index, self-reported average weekly alcohol consumption (expressed in standard glasses), smoking status, chronic liver disease, chronic heart failure, diabetes mellitus, active cancer, and polypharmacy [23–25].

We examined the association between educational level and time to first recurrent VTE and major bleeding using competing risk regression models according to Fine and Gray, accounting for overall death as a competing event [26]. The strength of the association between the educational level and clinical outcomes in the Fine–Gray model is reflected by the sub-hazard ratio (SHR), which is the ratio of hazards associated with the cumulative incidence function in the presence of a competing risk. For recurrent VTE, we adjusted for variables that were previously shown to be associated with recurrent VTE, including age, gender, body mass index, localization of the index VTE (PE with or without concomitant DVT, proximal DVT only, distal DVT only), type of VTE (provoked, unprovoked, or cancer-related), history of prior VTE, inflammatory bowel disease, and periods of anticoagulation as a time-varying covariate [27–34]. For major bleeding, we adjusted for variables that were previously associated with anticoagulation-related bleeding complications, including age, gender, self-reported average weekly alcohol consumption (expressed in standard glasses), overt pulmonary embolism, history of major bleeding, recent major surgery, cerebrovascular disease, chronic heart failure, diabetes mellitus, arterial hypertension, active cancer, chronic liver disease, chronic renal disease, risk of falls, polypharmacy, concomitant antiplatelet therapy, anemia, low platelet count, and periods of anticoagulation as a time-varying covariate [35–43].

We assumed missing values (see Table 1) in covariates used for adjustment to be normal or absent. All analyses were performed using Stata 14.0.

**Table 1:** Patient baseline characteristics by educational level.

	**Less than high school (N = 460)**	**High school (N = 206)**	**Post-secondary (N = 151)**	
**Characteristic**^a^	**n (%) or median (interquartile range)**			***P*-value**
Age, years	75 (69–82)	75 (69–79)	74 (69–81)	0.42
Female gender	242 (53)	98 (48)	41 (27)	<0.001
Living status				0.14
Living at home with someone else	274 (60)	142 (69)	98 (65)	
Living at home alone	174 (38)	58 (28)	51 (34)	
Living in a nursing home	12 (3)	6 (3)	2 (1)	
Localization of index VTE				0.79
PE (with/without DVT)	323 (70)	149 (72)	111(74)	
Proximal DVT	106 (23)	41 (20)	32 (21)	
Distal DVT only	31 (7)	16 (8)	8 (5)	
Type of index VTE				
Provoked^b^	108 (23)	42 (20)	34 (23)	0.68
Unprovoked^c^	317 (69)	136 (66)	103 (68)	0.76
Cancer-related^d^	35 (8)	28 (14)	14 (9)	0.05
Arterial hypertension	298 (65)	136 (66)	92 (61)	0.59
Diabetes mellitus	72(16)	32 (16)	24 (16)	1.0
Smoking status				0.49
Current smoker	29 (6)	14 (7)	13 (9)	
Past smoker	177 (39)	80 (39)	67 (44)	
Never smoker	245 (55)	112 (54)	71 (47)	
Body mass index (kg/m^2^)	27.3 (24.6–30.5)	26.9 (23.9–30.2)	26.6 (23.8–29.4)	0.03
Chronic heart failure^e^	36 (8)	13 (6)	10 (7)	0.75
Cerebrovascular disease^f^	42 (9)	20 (10)	11 (7)	0.71
Chronic pulmonary disease^g^	66 (14)	29 (14)	15 (10)	0.37
Chronic liver disease^h^	8 (2)	2 (1)	0 (0)	0.22
Chronic renal failure^i^	82 (18)	41 (20)	30 (20)	0.76
Inflammatory bowel disease	13 (3)	9 (4)	4 (3)	0.53
Prior VTE	128 (28)	67 (33)	48 (32)	0.39
History of major bleeding^j^	41 (9)	22 (11)	12 (8)	0.64
Standardized alcoholic drinks/week^k^	1 (0–7)	2 (0–7)	3 (0–7)	0.02
High risk of falls^l^	218 (47)	84 (41)	61 (40)	0.15
	**Less than high school (N = 460)**	**High school (N = 206)**	**Post-secondary (N = 151)**	
**Characteristic**^a^	**n (%) or median (interquartile range)**			***P*-value**
Anemia^m^	164 (36)	67 (33)	44 (29)	0.26
Platelet count <150 G/l	61 (13)	24 (12)	26 (17)	0.30
Serum creatinine >1.5 mg/dl	50 (11)	15 (7)	18 (12)	0.24
Antiplatelet/NSAID therapy^n^	160 (35)	96 (47)	64 (42)	0.01
Polypharmacy^o^	228 (50)	96 (47)	77 (51)	0.68
VKA therapy prior to VTE diagnosis	18 (4)	11 (5)	8 (5)	0.63
Type of initial parenteral anticoagulation				<0.001
Unfractionated Heparin	153 (33)	79 (38)	49 (32)	
Low molecular weight Heparin	204 (44)	101 (49)	69 (46)	
Fondaparinux	97 (21)	18 (9)	23 (15)	
Danaparoid	0 (0)	0 (0)	1 (1)	
No parenteral anticoagulation	6 (1)	8 (4)	9 (6)	
Use of inferior vena cava filter	4 (1)	1 (0)	1 (1)	0.86
Thrombolysis	14 (3)	6 (3)	5 (3)	0.98
Thromboembolectomy	0 (0)	0 (0)	2 (1)	0.01

VTE, venous thromboembolism; PE, pulmonary embolism; DVT, deep vein thrombosis; NSAID, non-steroidal anti-inflammatory drug; VKA, vitamin K antagonists.

^a^Data were missing for anemia (7%), platelet count (7%), and creatinine (8%).

^b^Major surgery, estrogen therapy, immobilization (fracture or cast of the lower extremity, bed rest >72 hours, or voyage in sitting position for >6 hours) during the last 3 months before index VTE.

^c^Absence of major surgery, estrogen therapy, immobilization, or active cancer during the last 3 months before index VT.

^d^Cancer requiring surgery, chemotherapy, radiotherapy, or palliative care during the last 3 months before index VT.

^e^Systolic or diastolic heart failure, left or right heart failure, forward or backward heart failure, or a known left ventricular ejection fraction of <40%.

^f^History of ischemic or hemorrhagic stroke with hemiparesis, hemiplegia, or paraplegia at the time of screening.

^g^Chronic obstructive pulmonary disease, active asthma, lung fibrosis, cystic fibrosis, or bronchiectasis.

^h^Liver cirrhosis, chronic hepatitis (B, C, autoimmune, etc.), chronic liver failure or hemochromatosis. Fatty liver was not considered a chronic liver disease.

^i^Chronic renal failure requiring or not hemodialysis such as diabetic or hypertensive nephropathy, chronic glomerulonephritis, chronic interstitial nephritis, myeloma-related nephropathy, or cystic kidney disease.

^j^Bleeding that led to a hospital stay or transfusions.

^k^Self-reported average weekly amount of alcoholic beverages during the last 12 months measured as standardized alcoholic beverages.

^l^Self-reported fall during the last year or any problem with gait, balance, or mobility.

^m^Hemoglobin <130 g/L for men and <120 g/L for women.

^n^Use of any antiplatelet therapy, such as aspirin, clopidogrel, prasugrel, aspirin/dipyridamol, or use of non-steroidal anti-inflammatory drugs.

^o^Prescription of >4 drugs, including St. John’s wort. The intake of vitamins or alternative medicine treatments was not considered.

## Results

### Study sample

**Fig 1:**
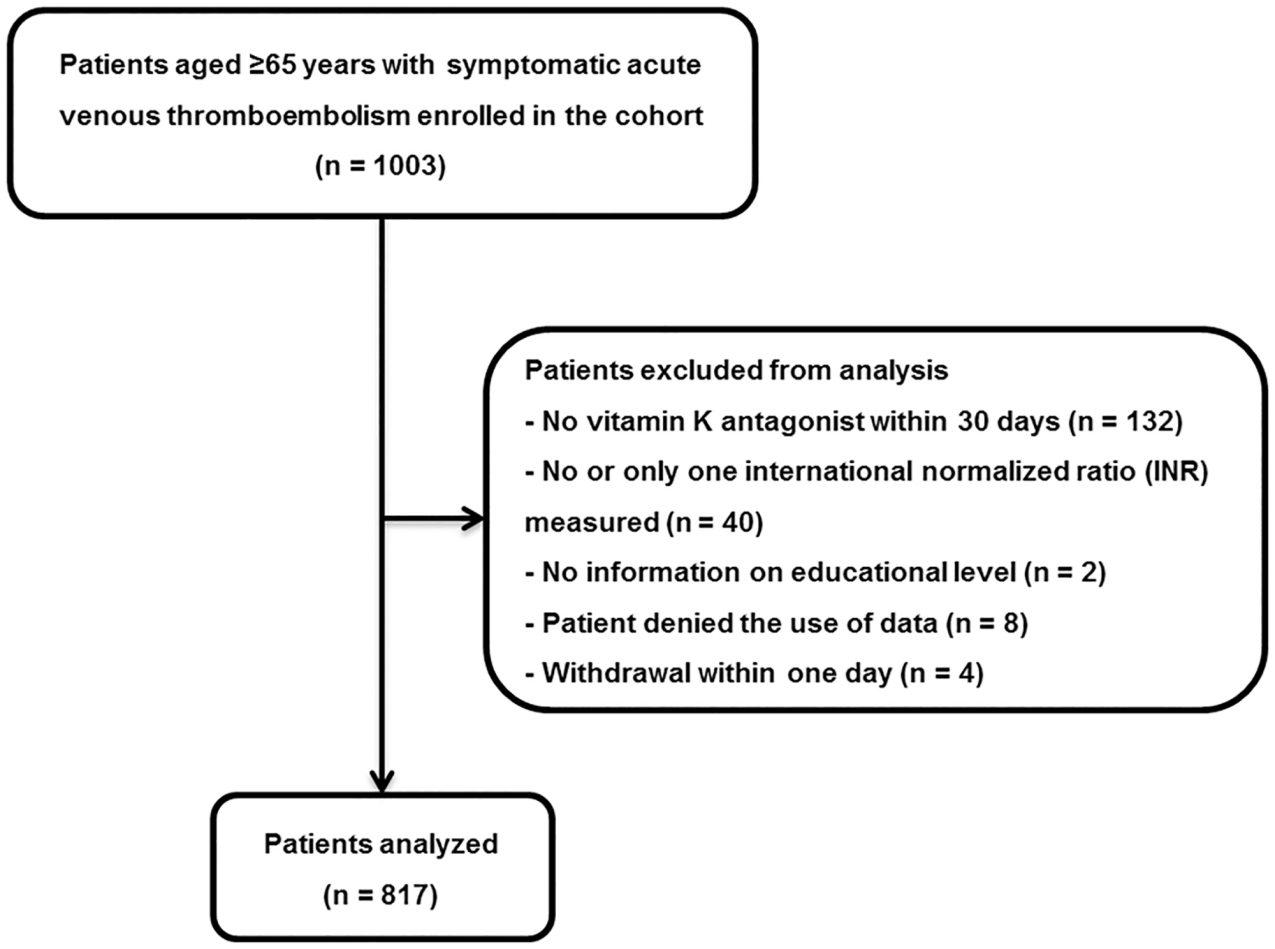
Patient flow chart.

Of the 1003 patients initially enrolled in the cohort [17], we excluded 186 patients, mainly patients with no initial oral anticoagulation (N = 132), leaving a final study sample of 817 patients with acute VTE (Fig 1). Excluded patients were more likely to be current or past smokers (56% vs. 47%, *P* = 0.01) and to have a high risk of falls (53% vs. 44%, *P* = 0.021), had a lower body mass index (median 25 vs. 27, *P*<0.001), had less often an unprovoked index VTE (24% vs.68%, *P*<0.001), and had more often active cancer (57% vs. 9%, *P*<0.001), a history of major bleeding (15% vs. 9%, *P* = 0.026), anemia (64% vs. 34%, *P*<0.001), and polypharmacy (58% vs. 49%, *P* = 0.016) than analyzed patients. Because direct oral anticoagulants were not yet authorized for treatment of acute VTE at the time of patient recruitment in Switzerland, none of the excluded patients was treated with direct oral anticoagulants.

Analyzed patients had a median age of 75.0 years (interquartile range [IQR] 69.0–81.0), 381 (47%) were women, and 556 (68%) had unprovoked index VTE. Overall, 460 patients (56%) had a less than high school education, 206 (25%) were high school graduates, and 151 (18%) had a post-secondary degree (Table 1). Patients with less than a high school education were more likely to be women and to have a higher body mass index, and were less likely to receive antiplatelet or non-steroidal anti-inflammatory drugs. They also had a lower alcohol consumption. The median follow-up period was 30 months (IQR 24–41).

### Educational level and quality of anticoagulation

There was no statistically significant difference in the percentage of TTR across the three educational levels, with a mean TTR of 61% (standard deviation [SD] 23%) in the less than high school group, 64% (SD 23%) in the high school group, and 63% (SD 21%) in the post-secondary group (*P* = 0.36, Table 2). The percentage of time above and below the therapeutic range did not differ by educational level. After adjustment for risk factors of poor anticoagulation control, measures of anticoagulation quality did not differ significantly between patients with less than high school education and those with a higher educational level (Table 3).

**Table 2:** Anticoagulation quality by educational level.

	Less than high school		High school	Post-secondary	
Anticoagulation quality	Mean percentage (SD)				*P*-value
Time in the therapeutic range (INR 2.0–3.0)	61.4 (22.7)	64.1 (23.3)		62.8 (20.9)	0.36
Time above the therapeutic range (INR >3.0)	15.0 (16.7)	14.9 (18.3)		15.1 (16.2)	0.99
Time below the therapeutic range (INR <2.0)	23.5 (22.0)	21.0 (20.8)		22.1 (19.5)	0.35

SD, standard deviation; INR, international normalized ratio.

**Table 3:** Association between educational level and anticoagulation quality.

Anticoagulation quality	Adjusted difference^a^ (95% CI)	*P*-value
	Percent	
**Time in the therapeutic range (INR 2.0–3.0)**		
Less than high school	Reference	-
High school	2.3 (-1.3 to 5.9)	0.21
Post-secondary	0.0 (-4.1 to 4.1)	1.0
**Time above the therapeutic range (INR >3.0)**		
Less than high school	Reference	-
High school	0.1 (-2.7 to 2.9)	0.95
Post-secondary	0.6 (-2.6 to 3.8)	0.71
**Time below the therapeutic range (INR <2.0)**		
Less than high school	Reference	-
High school	-2.4 (-5.9 to 1.1)	0.18
Post-secondary	-0.6 (-4.6 to 3.3)	0.75

INR, international normalized ratio; CI, confidence interval.

^a^Adjusted for age, gender, living status, smoking status, body mass index, alcohol consumption, chronic liver disease, history of heart failure, diabetes mellitus, active cancer, and polypharmacy.

### Educational level and clinical events

**Fig 2:**
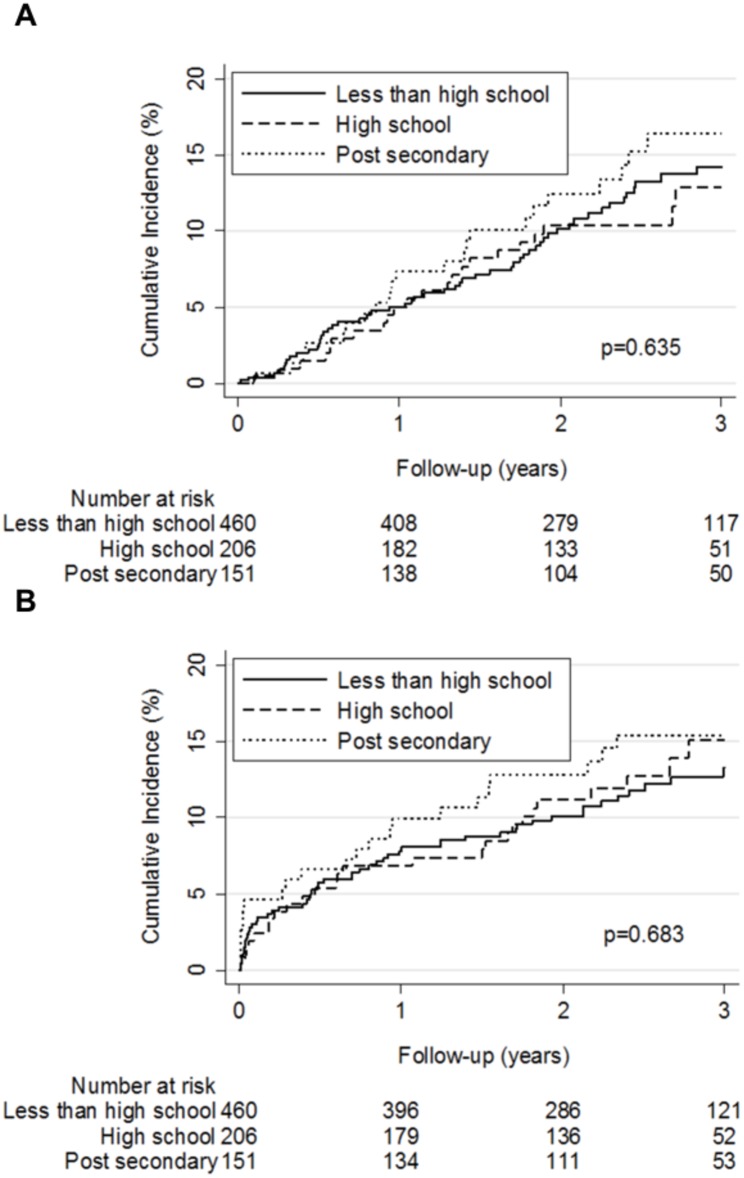
Kaplan-Meier estimates of clinical outcomes by educational level. Panel A. Kaplan-Meier estimates of a first recurrent venous thromboembolism by educational level. The 3-year cumulative incidence of a first recurrent venous thromboembolism was 14.2%, 12.9%, and 16.4% for patients with less than high school, high school, and a post-secondary degree, respectively (*P* = 0.64 by the logrank test). Panel B. Kaplan-Meier estimates of a first major bleeding by educational level. The 3-year cumulative incidence of a first major bleeding was 13.3%, 15.1%, and 15.4% for patients with less than high school, high school, and a post-secondary degree, respectively (*P* = 0.68 by the logrank test).

Overall, 110 patients (13.5%) died during follow-up. 105 patients (12.9%) had a first recurrent VTE and 102 (12.5%) had a first major bleeding during follow-up. The 3-year cumulative incidence of recurrent VTE and major bleeding did not differ across the three educational levels (Fig 2A and 2B). After adjustment, patients with a high school (SHR 0.95, 95% CI 0.56–1.61) or a post-secondary degree (SHR 1.14, 95% CI 0.68–1.92) did not have a lower risk of recurrent VTE compared to patients with less than a high school education. Similarly, patients with a high school (SHR 1.12, 95% CI 0.70–1.81) or a post-secondary degree (SHR 1.40, 95% CI 0.82–2.38) did not have a lower risk of major bleeding than patients with less than high school education (Table 4).

**Table 4:** Association between educational level, recurrent venous thromboembolism, and major bleeding.

	**Adjusted SHR**^a^ **(95% CI)**	***P*-value**
**Recurrent VTE**		
Less than high school	Reference	-
High school	0.95 (0.56–1.61)	0.85
Post-secondary	1.14 (0.68–1.92)	0.62
	**Adjusted SHR**^b^ **(95% CI)**	***P*-value**
**Major Bleeding**		
Less than high school	Reference	-
High school	1.12 (0.70–1.81)	0.63
Post-secondary	1.40 (0.82–2.38)	0.22

VTE, venous thromboembolism; SHR, sub-hazard ratio; CI, confidence interval.

^a^Adjusted for age, gender, body mass index, type of the index VTE, localization of the index VTE, history of prior VTE, inflammatory bowel disease, and periods of anticoagulation as a time-varying covariate.

^**b**^Adjusted for age, gender, alcohol consumption, overt pulmonary embolism, history of major bleeding, recent major surgery, cerebrovascular disease, chronic heart failure, diabetes mellitus, arterial hypertension, active cancer, chronic liver disease, chronic renal disease, risk of falls, polypharmacy, concomitant antiplatelet therapy, anemia, low platelet count, and periods of anticoagulation as a time–varying covariate.

## Discussion

In our prospective cohort of elderly patients with VTE, we found no association between the level of education and the quality of anticoagulation, recurrent VTE, or major bleeding. Our results are consistent with prior studies that did not demonstrate a relationship between the level of education and anticoagulation quality in mixed samples including patients with atrial fibrillation, VTE, and mechanical heart valves [11–13]. Although patients with a lower educational level have a limited language proficiency, a lower health literacy, and a poorer knowledge of anticoagulation therapy [5–7], a lower level of education does not appear to translate into a worse quality of anticoagulation or outcomes in elderly patients with VTE. Overall, our results indicate that elderly patients with VTE who have a low educational level do not need to be specifically targeted for intensified anticoagulation-related educational measures or surveillance.

In contrast to our findings, a study of elderly patients with atrial fibrillation reported that patients with a university degree spent more time in the therapeutic INR range [10]. Similarly, patients with atrial fibrillation who had a low income were more likely to be hospitalized for bleeding or to experience fatal bleeds [44]. A possible explanation is that the effect of educational level and other socioeconomic factors on anticoagulation quality may be more relevant in primary (e.g., stroke prevention in atrial fibrillation) than in secondary prevention (e.g., prevention of recurrent VTE) [45].

Somewhat paradoxically, a higher educational level was associated with a decreased adherence to warfarin in a prior study, possibly as a consequence of independent decision making or reduced trust in physicians relative to less educated patients [14]. However, this study evaluated the adherence to warfarin therapy, measured by electronically monitored pill bottle openings, and did not determine the TTR, a more direct indicator of anticoagulation quality.

Our study has several strengths. First, our prospective cohort enrolled in- and outpatients with acute VTE from nine Swiss university and non-university hospitals, increasing the generalizability of our findings. Second, we directly and objectively assessed anticoagulation quality using the TTR rather than self-reported or electronically measured anticoagulation compliance. Third, clinical outcomes, such as recurrent VTE and major bleedings, were adjudicated by a committee of three blinded clinical experts using pre-defined criteria, reducing the risk of detection bias. Finally, to decrease the risk of confounding, our analyses were adjusted for the majority of known risk factors of poor anticoagulation control, recurrent VTE, and major bleeding.

Our study has potential limitations. First, our study enrolled exclusively patients aged 65 years or older with acute VTE. We thus cannot generalize our results to younger patients or those with other indications for anticoagulation. Because patients were enrolled exclusively in hospital in- and outpatient services, healthier patients with milder forms of VTE (typically DVT) who are managed in private practices may be underrepresented in our study. Second, the level of education was self-reported in our study, which may have resulted in an overestimation of the educational level in some patients [46]. Third, we could not evaluate other socioeconomic factors with known impact on anticoagulation quality and outcomes, such as patient income and living area [23, 44, 47–49]. However, Swiss residents have universal health care coverage and a good access to health care, including anticoagulant drugs and monitoring [50]. Moreover, there was no relationship between income class and access/affordability of vitamin K antagonists in an international study [51]. Fourth, patients with severe dementia and those with insufficient language skills were not enrolled in our cohort, both risk factors for poor anticoagulation control [5, 23]. Thus, we cannot exclude the possibility that the inclusion of such patients would have influenced our results. Finally, we used the TTR as a measure of anticoagulation quality. Although it is associated with drug adherence [52, 53], other factors such as comorbid conditions, variations in food intake or drug interactions may also have influenced the TTR.

## Conclusion

In conclusion, our results did not show an association between educational level and anticoagulation quality or clinical outcomes in elderly patients with acute VTE who were treated with vitamin K antagonists. Our findings indicate that elderly patients with VTE who have a low educational level do not need to be specifically targeted for intensified anticoagulation-related educational measures or surveillance.
